# The cell cycle and pluripotency

**DOI:** 10.1042/BJ20121627

**Published:** 2013-03-28

**Authors:** Christopher Hindley, Anna Philpott

**Affiliations:** *Emmy Noether-Group for Stem Cell Biology, Department of Molecular Embryology, Institute of Anatomy and Cell Biology, University of Freiburg, Freiburg, D-79104, Germany; †Department of Oncology, University of Cambridge, Hutchison/Medical Research Council (MRC) Research Centre, Cambridge CB2 0XZ, U.K.

**Keywords:** cell cycle, Myc, pluripotency, S-phase, Cdc, cell division cycle, CDK, cyclin-dependent kinase, CDK1, CDK inhibitor, dKO, double knockout, ESC, embryonic stem cell, hESC, human ESC, iPSC, induced pluripotent stem cell, KO, knock out, MEF, mouse embryonic fibroblast, mESC, murine ESC, miRNA, microRNA, PSC, pluripotent stem cell, R, restriction point, Rb, retinoblastoma, shRNA, short hairpin RNA

## Abstract

PSCs (pluripotent stem cells) possess two key properties that have made them the focus of global research efforts in regenerative medicine: they have unlimited expansion potential under conditions which favour their preservation as PSCs and they have the ability to generate all somatic cell types upon differentiation (pluripotency). Conditions have been defined *in vitro* in which pluripotency is maintained, or else differentiation is favoured and is directed towards specific somatic cell types. However, an unanswered question is whether or not the core cell cycle machinery directly regulates the pluripotency and differentiation properties of PSCs. If so, then manipulation of the cell cycle may represent an additional tool by which *in vitro* maintenance or differentiation of PSCs may be controlled in regenerative medicine. The present review aims to summarize our current understanding of links between the core cell cycle machinery and the maintenance of pluripotency in ESCs (embryonic stem cells) and iPSCs (induced PSCs).

## THE CELL CYCLE IN PLURIPOTENT CELLS

The eukaryotic cell cycle refers to the series of events comprising the sequential actions, during proliferation, of synthesis of DNA (S-phase) and cell division (M-phase) with intervening gap phases to allow cell growth (G_1_-phase, after M- but before S-phase) and to check the integrity of genomic material (G_2_-phase, after S- but before M-phase). The cell cycle is regulated at the molecular level by the phase-specific activity of a series of CDKs (cyclin-dependent kinases) and made irreversible by the regulated degradation of cyclin subunits (for reviews see [1,2]). The early G_1_-phase is characterized by the activity of the D-type cyclins and CDKs 4 and 6. During G_1_, cells are responsive to extracellular signalling and, in particular, mitogenic signalling through such pathways as the MAPK (mitogen-activated protein kinase) pathway, which regulates the activity of cyclin D and/or CDK4/6. The action of cyclin D and CDK4/6 drives progression through G_1_ past a point known as the restriction point (R). Progression beyond R means that the cell no longer requires mitogenic signalling and is committed to DNA synthesis, chromosome segregation and cytokinesis. Thus cells are responsive to proliferation or differentiation cues during G_1_ and integrate these signals when deciding whether to commit to cell division (pass R) or to withdraw from the cell cycle and differentiate.

In late G_1_, cyclin D–CDK4/6 activity begins to decrease and cyclin E–CDK2 activity rises. Cyclin E–CDK2 has a number of important targets for promoting cell cycle progression, in particular the Rb (retinoblastoma) protein which inhibits the E2F transcription factor when hypophosphorylated. As G_1_ progresses, phosphorylation of Rb protein increases, relieving its inhibition of E2F which ultimately allows E2F to up-regulate a number of targets important for S-phase entry and progression. During early S-phase, cyclin E is degraded and cyclin A complexes with CDK2 to drive progression through S-phase and into G_2_. From mid-G_2_ onwards, the activity of CDK2 decreases and cyclin A associates with CDK1 [formerly known as Cdc2 (cell division cycle 2)]. Finally, at entry to M-phase, cyclin B complexes with CDK1 to phosphorylate a number of targets involved in nuclear envelope breakdown, chromosome condensation, and segregation and cytokinesis. Degradation of cyclin B following cytokinesis signifies the start of the next G_1_.

*In vivo* data on the overall cell cycle structure of mammalian ESCs (embryonic stem cells) were obtained over 30 years ago, although molecular detail has only been uncovered more recently with the development of techniques to culture PSCs (pluripotent stem cells) *in vitro*. Cell cycling in rodent ESCs is rapid, estimated at 8–10 h [3,4] and is assumed to be similar to that of peri-implantation embryos. Generation time decreases further with a burst of proliferation following implantation and prior to gastrulation, with the average division time being estimated at 4.5–8 h [4]. Such rapid cell cycling is an effect of the unusual cell cycle structure of ESCs compared with that of somatic cells. ESCs have truncated gap phases, and an unusually high proportion of asynchronously dividing cells are in S-phase (~65%) when compared with G_1_ (~15%). Interestingly, ESCs are also small in size when compared with somatic cells, a feature that is often attributed to a shortened period of growth in the truncated G_1_-phase (reviewed in [5]). It has been demonstrated that the level of cyclins and the activity of CDKs oscillates with a lesser amplitude in mESCs (murine ESCs) than in somatic cells due to a high level of expression of the APC/C (anaphase-promoting complex) inhibitor Emi1 [6]. This study is in contrast with earlier work which suggested that cyclins and CDKs do not oscillate during cell cycle progression in mESCs. Upon differentiation, the cell cycle is restructured such that approximately 40% of an asynchronously dividing population of cells are found in G_1_ [3,7].

hESCs (human ESCs) are similar to mESCs in that S-phase is highly populated (~50% of cells) and *cyclin E* (HUGO approved symbol *CCNE*) expression does not display periodicity, but is constitutive [8]. Also similar to mESCs, *cyclin A* (HUGO approved symbol *CCNA*) expression is found to oscillate in hESCs and, although no formal assays of CDK2 activity were undertaken, the presence of hypophosphorylated Rb specifically in G_1_-phase suggests that CDK2 activity is subject to cell cycle regulation. hESCs display a longer generation time (15–16 h; reviewed in [9,10]) than mESCs, suggesting that the overall time taken to divide is not a crucial regulator of pluripotency.

Comparisons can be drawn between the cell cycle of mammalian ESCs and that of the early embryos of both invertebrate and anamniote species, such as *Xenopus laevis*. Because of the more easily accessible nature of *Xenopus* embryos, molecular details of the cell cycle in these early embryonic cells were obtained far earlier. In *Xenopus* embryos, maternal stockpiles of mRNA and proteins drive the cell cycle prior to the onset of zygotic transcription, and the cycle lacks gap phases, instead oscillating between S- and M-phases [11]. This minimal cell cycle is responsible for the rapid and synchronous division seen in early-stage invertebrate and anamniote embryos and is driven by alternating CDK2 (S-phase) and Cdc2 (M-phase) activities. After zygotic transcription begins, the cell cycle lengthens to include G_1_- and G_2_-phases [12]. Although cell division is still rapid and widespread, cell fate becomes restricted and in addition cyclins and CDKs display tissue-specific patterns of expression [13]. These data are consistent with our general metazoan model that differentiation requires a G_1_-phase for the integration of differentiation signals and suggests that cell cycle components may play roles beyond simply driving cell proliferation. Indeed, eukaryotic cells require only oscillating cyclin B–Cdc2 activity in order to undergo full cell cycling [14,15]. If the regulation of Cdc2 activity is necessary and sufficient for a minimal cell cycle, this implies that other cyclin–CDK combinations may have additional roles [16]. For instance, *Xenopus* embryos do not express D-type cyclins strongly until relatively late in development, well after the establishment of gap phases, and only then to a significant level in the developing eye [13,17].

**Figure 1:**
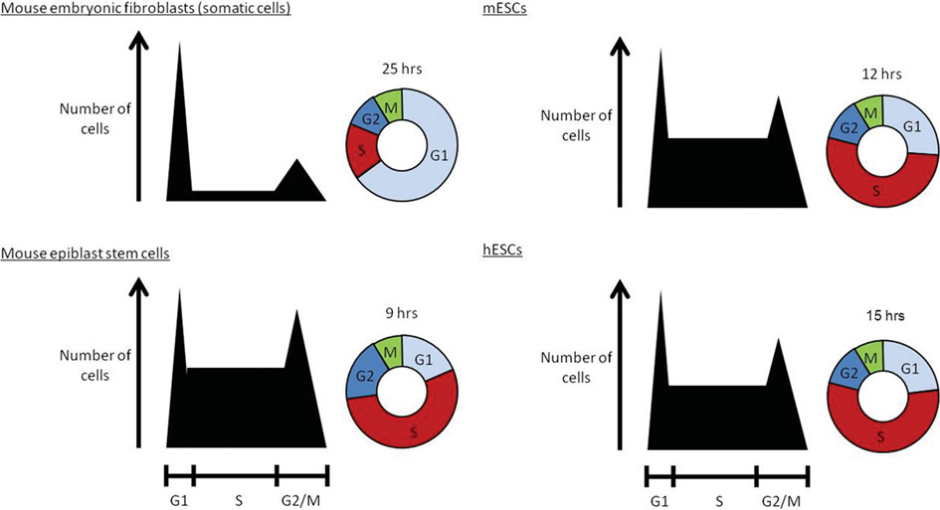
Schematic diagram comparing the cell cycle in somatic (MEF) and pluripotent cells For each panel, the first part is a graphical representation of the number of cells in each phase of the cell cycle within a population, as assessed by propidium iodide staining and flow cytometric analysis. Peaks represent 2N and 4N DNA content. The second part of each panel is a summary for an individual cell of the relative amounts of time spent in each cell cycle phase. In addition the average total time taken to complete one cycle is presented for each cell type. It is clear that, proportionally, pluripotent cells have a shortened G_1_- and a longer S-phase for each cycle than somatic cells, although absolute S-phase length is comparable.

The cell cycle with truncated gap phases is a feature of both rodent and human ESCs (see Figure 1), although differences in the regulation of cyclin–CDKs are explored in more detail below. Such differences may be a result of miscomparison, as hESCs are now believed to be more similar to rodent epiblast stem cells than to rodent ESCs [7]. The explanation of such differences is part of a general trend towards the description of differences at the population level as different ‘flavours’ of pluripotency [18], whereas investigation at the single-cell level suggests that a population of PSCs is, in fact, a collection of metastable pluripotent states that, at the population level, then exhibits the recognizable properties of both self-renewal and spontaneous differentiation (reviewed in [19,20]). A recent study has demonstrated that murine PSCs cycle into and out of the pluripotent and totipotent states [21]. In the light of the revelation of such heterogeneity within PSC populations, it would presumably be fruitful to investigate processes which could act to homogenize the functional outcomes of such heterogeneity and thus lead to the reproducible sequence of events seen during normal development.

## THE CELL CYCLE AND THE REGULATION OF PLURIPOTENCY

The studies described above suggest that rapid cell cycling is a feature of PSCs, but, given the discrepancy between hESCs and mESCs in the time taken to divide, that the length of the cell cycle is not a determinant of the level of pluripotency. Do specific cell cycle components regulate pluripotency at all? A recent study of the hESC phospho-proteome during differentiation revealed that CDK2 and Cdc2 activities were central in promoting pluripotency and self-renewal [22]. This is in agreement with earlier studies which have highlighted *CDK2* as having a specific role in the maintenance of pluripotency. Use of the broad-spectrum CDK1 (CDK inhibitor) roscovitine in hESC culture promoted G_1_/S arrest, accumulation of hypophosphorylated Rb, smaller hESC colonies and the down-regulation of the pluripotency marker *Oct4* [3]. It is possible then that CDK2 activity serves as a regulator of the earliest restrictions in cell fate. Activation of p53 in hESCs by the small molecule nutlin leads to the degradation of cyclins A and E, and the inactivation of CDK2 [23]. This results in G_1_/G_0_ arrest and rapid differentiation of the hESCs. Although the p53 target *p21* was up-regulated during CDK2 inactivation, its involvement in the increased differentiation that was observed was unclear.

In addition to a role maintaining the early pluripotency of ESCs, CDK2 activity has also been implicated in cell-fate decisions taken during later embryogenesis. The transcription factor Cdx2 has been identified as a direct target of CDK2 and the phosphorylation of Cdx2 by CDK2 promotes its degradation and inhibits differentiation in the intestine [24]. A further hESC study confirmed the cell-cycle-dependence of CDK2 activity in hESCs and demonstrated that specific knockdown of *CDK2* using siRNA (small interfering RNA) induced arrest in G_1_ and differentiation of hESCs to extra-embryonic lineages [25]. Although this study lacked the population characterization of the previous study (which ruled out contaminant differentiating cells from analysis), its results are consistent with a study showing that the CDK2 inhibitor CDK2-associating protein 1 was required for promoter methylation and down-regulation of *Oct4* during differentiation [26]. As this result was obtained in mESCs, this supports a role for *CDK2* in controlling pluripotency in mESCs as well as hESCs [27].

CDK2 activity may not, however, be a direct regulator of pluripotency. In a study of cell cycle regulation in mESCs, Stead et al. [3] demonstrated that only cyclin B–Cdc2 activity was regulated in a cell-cycle-dependent manner, i.e. that cyclin B–Cdc2 activity was high during late G_2_/M-phases and low during G_1_/S-phases [3]. The cyclins A2 and E1 were found to be constitutively expressed at levels exceeding those seen in somatic cell lines. As a result, although *Cdk2* expression was comparable with somatic cell lines, it was active throughout the cell cycle and displayed no cell-cycle-dependent regulation in this study. Although CDK2 activity did not oscillate with the cell cycle phase, as seen in somatic cells, the activity was not maximal and could be increased by treatment with the cell cycle phosphatase Cdc25b. Inhibitory tyrosine phosphorylation, reversed by Cdc25b overexpression, appears to be the only mechanism controlling CDK2 activation here, as expression of CDKIs of the *Cip* (CDK-interacting protein)/*Kip* (kinase inhibitory protein) family was not detected. Treatment with a specific CDK2 inhibitor, Ro09-3033, increased the generation time of mESCs by 66% but, contrary to expectations, did not alter the cell cycle structure as all phases were lengthened proportionally. mESCs also continued to express the pluripotency markers *Rex-1* and *Oct4* following treatment with Ro09-3033. Altogether, this suggests that the cell cycle of mESCs owes its rapidity to a constitutive level of CDK2 activity, but that cell cycle structure and pluripotency are not themselves controlled by CDK2 activity [11,13].

The results described above suggest that CDK2 does not directly regulate pluripotency. Rather, it may be the other way around as one study has shown that the key pluripotency regulator NANOG controls entry to S-phase in hESCs by promoting the expression of Cdc25C and CDK6 [28]. So perhaps the key determinant of pluripotency is the short time spent in G_1_- relative to S-phase? This would be consistent with the results of the small-molecule inhibitor treatments on pluripotency: roscovitine, which prevents S-phase entry and so increases the length of time spent in G_1_, inhibits the pluripotent state, whereas Ro09-3033, which lengthens G_1_-, S- and G_2_-phases proportionally by inhibiting CDK2, is permissive for it despite slowing overall cell division [3]. This result echoes the observation that hESCs divide relatively slowly, especially when compared with their murine counterparts, and therefore the overall length of the cell cycle is unlikely to be a critical determinant of pluripotency. Conversely, an increase in the length of time spent in G_1_- relative to S-phase could be the trigger for differentiation. As it is believed that *in vivo* the length of S-phase in PSCs is not significantly different from that of somatic cells [29], this would suggest that the length of G_1_ is critical for regulating pluripotency. From the data outlined above, however, exogenous manipulations of the length of S-phase could produce the same effect.

Some studies have highlighted the presence of hypophosphorylated Rb during differentiation promoted by CDK2 inactivation in hESCs [8,26]. In PSCs, a major regulator of the length of G_1_ is Rb, which functions as an inhibitor of the E2F transcription factor and so is responsible for inhibiting the G_1_/S-phase transition. The presence of the hypophosphorylated form of Rb, which is active as an E2F inhibitor, correlates with differentiation potential in hESCs [8] and only the constitutively phosphorylated pRb form, which cannot inhibit E2F, is found in mESCs [3]. By controlling the length of G_1_ relative to the other cell cycle phases, the phosphorylation status of Rb may be crucial to determining the overall structure of the cell cycle. However, experiments directly manipulating the length of G_1_ relative to S-phase have yet to be performed and so there is, as yet, no direct evidence that the G_1_/S-phase ratio is a crucial determinant of pluripotency.

## THE ROLE OF S-PHASE IN PLURIPOTENCY

How might a relatively long time spent in S-phase stabilize the pluripotent state? The crucial activity of S-phase, DNA replication, presents a unique opportunity during the cell cycle for the genetic and epigenetic regulation that may be involved in stabilizing the pluripotent state. One possibility is that the proteins directly regulating DNA replication might also stabilize pluripotency. The protein geminin regulates the loading of MCM (mini-chromosome maintenance) proteins on to replication origins, and the availability of geminin thus regulates the replication of DNA during S-phase and is a major control to prevent endoreplication during M-phase (reviewed in [30–32]). *Geminin* (HUGO approved symbol *GMNN*) is down-regulated in trophoblast giant cells and the ablation of *geminin* is sufficient to cause commitment of pluripotent inner cell mass cells to the trophoblast lineage [33]. In addition to its role in preventing endoreplication, geminin is present in G_1_ in mESCs and directly maintains the expression of the pluripotency genes *Sox2*, *Nanog* and *Oct4* [34]. More recently, roles for *geminin* in the regulation of lineage and stem cell properties during haemopoiesis have also been identified [35,36].

In addition to direct regulation of pluripotency by the DNA replication machinery, it is possible that the higher-order chromatin rearrangements that also occur during S-phase in order for DNA replication to proceed could provide epigenetic regulation that stabilizes the pluripotent state. An intensive investigation of epigenetic regulation in PSCs has revealed that PSCs have a much greater percentage of their genome in the open euchromatic state than somatic cells (reviewed in [37]), exhibit a unique pattern of gene methylation [38] and mark histones of key developmental regulator gene promoters with a bivalent pattern of both activating H3K4me^3^ (histone H3 Lys^4^ trimethylation) and repressive H3K27me^3^ (histone H3 Lys^27^ trimethylation) marks [39]. Each of these characteristics contributes to the transcriptional network that maintains pluripotency. Such epigenetic regulation results in a transcriptionally hyperactive ‘leaky’ genome [40]. Such a ‘leaky’ state could account for the dynamic heterogeneity seen at the single-cell level in PSC cultures [21] and highlights the importance of transcription factor networks in stabilizing or destabilizing the pluripotent state, prior to the establishment of heterochromatic silenced regions during development. Direct interactions have been reported between CDKs and epigenetic regulators involved in the maintainence of pluripotency, such as the DNA methylase DNMT1 [41] and the higher-order chromatin organizer HP1α [42], but it is unclear as to how these interactions regulate pluripotency and development.

**Figure 2:**
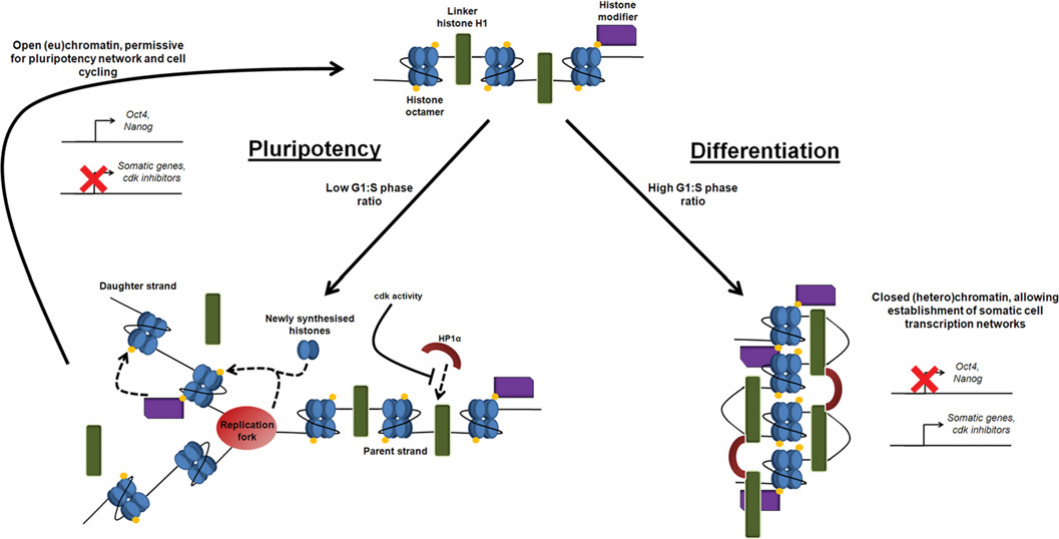
Schematic diagram of how S-phase may maintain the euchromatic state in PSCs A short G_1_/S-phase ratio results in more frequent passage of the replication fork, and although histone octamers and their modifications are conserved, linker histone H1 binding to DNA is not (left-hand side). This inhibits the formation of higher-order chromatin structure, which is then permissive once the G_1_/S-phase ratio increases (right-hand side).

As DNA replication requires chromatin to be in an open state, it is conceptually easy to see how a short G_1_ relative to S-phase could prevent the accumulation of heterochromatin. The maintenance of widespread euchromatin could be driven mechanistically by negative regulation of linker histone H1 activity. The linker histone H1 variants (there are 11 family members in humans [43]) intercalate between the standard histone octamers, composed of a dimer of tetramers of the histones H2A, H2B, H3 and H4, and so initiate areas of higher-order heterochromatin structure [44]. Consistent with increased higher-order chromatin in somatic cells, histone H1 transcription is seen to increase during development and genetic ablation of histone H1c, d and e is sufficient to stabilize mESCs in the pluripotent state and in particular to inhibit differentiation along the neuroectodermal lineage [45]. It is likely that CDK activity itself directly regulates the ability of histone H1 to bind to DNA, as the import of histone H1 into the nucleus has been reported to be inhibited by CDK activity [46]. However, the same report also suggests that histone H1 binding to DNA is facilitated by CDK1 activity, suggesting that the regulation of subcellular localization is responsible for appropriate histone H1 activity only during M-phase. In addition, although the transcription of the standard histone octamer components is cell-cycle-dependent [47] and loading of these histones on to newly synthesized daughter DNA strands is carried out by components associated with the replication fork {e.g. CAF-1 (chromatin assembly factor 1) [48]}, there is no evidence that histone H1 loading is synchronized with the cell cycle. Therefore it is possible that the passage of the replication fork serves to dissociate histone H1 from DNA and so prevents stable interaction leading to the formation of heterochromatin (see Figure 2), although to the best of our knowledge this mechanism linking the frequency of S-phase entry to pluripotency has not yet been directly investigated.

Although it is easy to imagine how the passage of the replication fork could prevent the formation of heterochromatin, thus stabilizing a pluripotent state, such a mechanism is, at first glance, at odds with the conservation through S-phase of histone and DNA modifications stabilizing the pluripotent state. As the passage of the replication fork would entail the disruption of complexes involved in regulating histone and DNA modification, and also promote the inclusion of freshly synthesized histones into the standard octamers, it might be expected that the unique patterns of histone and DNA modification would be attenuated with an increased rate of S-phase entry. The established model of histone formation on the daughter strands of DNA involves the direct recycling of parental histones on to the freshly replicated DNA (reviewed in [49,50]), thus allowing conservation of histone modifications between the parental and both daughter strands by ‘priming’ each daughter strand with half of the modified histones of the parental chromatin. A recent report [51] has challenged this model by providing evidence that it is the histone modifiers, in this case the *Drosophila* histone methyltransferases Trx and Pc, which are stably transmitted to the daughter strands from the parent strand. This report also provided evidence that histone modifications are lost from parental histones during passage of the replication fork, making the transfer of histone modifiers to the daughter strands the prime mechanism for conservation of histone modification through S-phase. In turn, it has also been suggested that incomplete transfer of histone modifications may inhibit cell cycle progression through G_1_, so preventing consistent erosion of histone modifications during S-phase [52]. Therefore, although the precise mechanism of conservation of histone modifications is uncertain, it would appear that such modifications are stable to the passage of the replication fork.

The evidence available suggests a model whereby increased S-phase entry and more frequent DNA replication would be permissive for the maintenance of epigenetic marks already present, but would prohibit the stable loading of histone H1 and interaction with epigenetic regulator complexes (Figure 2). This would prevent the formation of heterochromatin and the establishment of somatic cell transcriptional programmes, thus stabilizing the pluripotent state. However, such a model suffers from a lack of direct investigation and, despite the undisputed importance of epigenetic regulation in the maintenance of the pluripotent state, there is a lack of data on how such regulation is maintained during the PSC cell cycle and how the cell cycle and epigenetic machineries interact.

## THE CELL CYCLE DURING SOMATIC CELL REPROGRAMMING

The ability to convert somatic cells into PSCs (reprogramming) has been a long-held goal of regenerative medicine. Although it has been possible to reprogramme somatic cell nuclei by transplantation into a suitable donor cytoplasm for decades [53–55], the discovery of four genes (*Oct4*, *Sox2*, *c-Myc* and *Klf4*; referred to hereafter as the four factors) whose overexpression could convert fibroblasts into iPSCs (induced PSCs) opens the door for reprogramming on a biomedically relevant scale [56]. Work performed on the mechanism of reprogramming using the four factors suggests that although conversion into the pluripotent state is stochastic [57,58], reprogramming itself follows a broadly defined timetable. Furthermore, the efficiency of reprogramming is correlated with the number of cell divisions undergone during the stochastic phase [58]. First, the somatic cell transcriptional programme is down-regulated, as assessed by the down-regulation of fibroblast-specific genes [59]. Secondly, the genome undergoes broad epigenetic modification to a more PSC-like state, including demethylation of promoters for pluripotency-associated transcription factors such as Oct4 and Nanog and reactivation of the inactive X chromosome [60,61]. Finally, a stable transcriptional network of pluripotency-associated factors leads to the establishment of full pluripotency. Interestingly, partially reprogrammed cells exist in a stable state, suggesting that the establishment of pluripotency is a defined state and that, similar to the dKO;*GATA6* KO (KO is knockout and dKO is double knockout) cells described below, cells may continue to survive and divide in the absence of both pluripotency and lineage specification [62]. This highlights the role of a stable transcriptional programme in providing a cell with an identity during development, perhaps best highlighted by the dedifferentiation seen when PAX5 is depleted from mature B-cells [63,64] and the fat conversion into muscle which takes place on the introduction of MyoD to fibroblasts [65,66].

So does the cell cycle influence the reprogramming process at all? It is highly likely, as it is generally observed that older or more slowly dividing cells are more difficult to reprogramme. In addition, a marked increase in the efficiency of reprogramming is observed when fibroblasts are permeabilized and incubated in meiotic *Xenopus* egg extract (with high CDK1 activity) following infection with the four factors [67]. This increase in efficiency is not observed following incubation in interphase (low CDK1 activity) egg extract. A previous review has highlighted the role of the cell cycle in reprogramming during somatic cell nuclear transfer [68], particularly the role of M-phase and M-phase cytoplasmic factors in this process. However, it is unlikely that exactly the same mechanisms operate during four-factor-mediated reprogramming. First, the dissociation of transcription factors and epigenetic regulators observed upon chromatin condensation could indeed be significant for down-regulating the somatic cell transcription network, but only in the context of a rapid cell cycle leading to a relatively higher amount of time spent in M-phase. While this would be consistent with the rapid cell division time of mESCs, it is inconsistent with the long cell division time of hESCs, and four-factor-mediated reprogramming is observed in cells from both species [56,69]. Secondly, epigenetic marks, particularly DNA methylation, are not erased during M-phase and are in fact the probable cause of the low efficiency of successful mammalian cloning following somatic cell nuclear transfer [68]. This is inconsistent with the known epigenetic changes that occur during four-factor-mediated reprogramming. However, it is known that cell cycle alteration from a somatic to a more PSC-like structure is one of the first events to occur during reprogramming [62] and may be rate-limiting for the reprogramming events [70].

It seems particularly important that cells accumulate in S-phase following a short G_1_ for reprogramming [70], but once again the mechanistic significance of a short G_1_ on the establishment and maintenance of pluripotency is unknown. Although links between cell-cycle-regulated epigenetic phenomena and pluripotency have been reported [71], it is not yet established that a decreased G_1_/S-phase ratio regulates epigenetic modifications during reprogramming. So, although most emphasis within the field has been placed on changes within transcription networks and epigenetic changes occurring during reprogramming, it would be enlightening to also address further the role of G_1_ contraction/S-phase lengthening.

A small number of studies have attempted to assess the role of the cell cycle in reprogramming directly. The *Ink4*/*Arf* locus is epigenetically silenced in PSCs and, upon reprogramming, the kinetics with which such silencing occurs suggest that it is among the earliest events in the establishment of pluripotency [72]. Furthermore, this study demonstrated that the locus is a barrier to reprogramming, as *Ink4*/*Arf* deficient MEFs (mouse embryonic fibroblasts) reprogramme with a 15-fold higher efficiency than wild-type MEFs. Such conclusions are consistent with a previous study demonstrating that the p53 tumour suppressor pathway is also a barrier to reprogramming [73]. Here, use of shRNA (short hairpin RNA) against the p53 target gene *p21^cip1^* allowed increased reprogramming efficiency at a level comparable with that achieved by the use of shRNA against *p53* itself. It is highly likely that the p53 pathway and CDKIs maintain a barrier to the pluripotent state by forming an interconnected regulatory network, as p53 up-regulates *p19^INK4d^*, which is also responsible for up-regulating *p21^cip1^* [72,73]. Although not formally assessed, given the role of CDKIs in lengthening G_1_ and preventing S-phase entry, it is likely that such a barrier is imposed as a result of being unable to remodel the cell cycle towards a PSC-like state.

A later study also addressed the roles of cyclin–CDKs in enhancing the reprogramming process and identifies *cyclin D1* and *CDK4* as factors that increase the efficiency of reprogramming [74]. The identification of this complex, as distinct from cyclin A or E together with CDK2, as promoting the pluripotent state is interesting considering the lack of D-type cyclin activity in mESCs and the constitutive activity of CDK2 in ESCs. An earlier study from the same group had also highlighted the role of cyclin D in enhancing reprogramming efficiency, but indirectly through up-regulation by the GTPase Rem2 [70]. Overexpression of Rem2 was found to enhance reprogramming efficiency with three factors 8-fold and to the same extent as the addition of c-Myc. However, direct overexpression of cyclin D1 only enhanced reprogramming efficiency 3-fold. The authors point out the dual regulation of cyclin D1 and p53 by Rem2 and also note that both regulate apoptosis [70,75]. It is therefore unclear as to whether cyclin D1 overexpression acts here to enhance reprogramming through its regulation of the cell cycle. However, there is a general trend outlined in these studies that the efficiency of reprogramming correlates with the activity of cyclin–CDK complexes in G_1_, supported by the data that the addition of CDKIs to reprogramming factors decreases the efficiency of reprogramming from the base level [74]. Why then the inconsistency with data on the cell cycle in ESCs, which emphasizes the role of cyclin A and CDK2? It is possible that the process of efficient reprogramming requires a different cell cycle structure to that required for the maintenance of pluripotency. Alternatively, iPSCs may differ from ESCs in their cyclin–CDK complex activity, but not in the overall structure of the cell cycle.

## THE ROLE OF Myc AS A MASTER REGULATOR OF PLURIPOTENCY

So far we have considered the direct ways in which cell cycle components, or overall cell cycle structure, regulate the pluripotent state. However, much of the data described above is also consistent with a model whereby a master regulator of both the PSC-like cell cycle and transcription factors associated with pluripotency co-ordinates the two. The existence of such a master regulator is not mutually exclusive of the idea of stabilization of the PSC cycle by the pluripotent transcription factor network and vice versa, but it would allow such a self-reinforcing state to transition more readily, as a master regulator could simultaneously regulate both components. It is clear from several studies that *N-* and *c-Myc* could function as just such master regulators.

The *Myc* family (*c-Myc*, *N-Myc* and *L-Myc*) are bHLH (basic helix–loop–helix) transcription factors which co-ordinate the up-regulation of genes associated with S-phase entry, and *c-Myc* mis-regulation has a long history of involvement in tumorigenesis (reviewed in [5,76]). The role of the *Myc* family in pluripotency has been reviewed in full in [5]. However, briefly, *c-Myc* forms a metastable pluripotent state in PSCs where the pluripotent state is destabilized [78]. *N-* and *c-Myc* are found to be functionally redundant during murine development [79], but in dKO murine PSCs, the loss of both factors leads to spontaneous differentiation [80]. dKO cells were found to have a shortened S-phase and lengthened G_1_- and G_2_/M-phases, consistent with the function of a relatively long S-phase in maintaining pluripotency.

The *Myc* family are likely to regulate the structure of the cell cycle in PSCs through the up-regulation of the *mir*-*17*-*92* miRNA (microRNA) cluster, which regulates the translation of the cell cycle components *E2F1*, *cyclin D1*, *p21* and *Rb2*/*p130*. Intriguingly, although *Myc* directly repressed the differentiation of PSCs to primitive endoderm via inhibition of *GATA6* transcription, it also appeared to be required for differentiation into other lineages, as dKO;*GATA6* KO cells displayed no identifiable lineage commitment despite the spontaneous loss of pluripotency. This would be consistent with *Myc* as a master regulator as described above, maintaining pluripotency but also poising the cell for differentiation. In this regard, it is interesting that *c-Myc* associates with both activating and repressive epigenetic modifiers as determined by co-immunoprecipitation [81].

Further to its role in the maintenance of pluripotency, c-Myc is also one of the four reprogramming factors. Within the broad reprogramming schedule, the roles of the individual four factors in reprogramming have been assessed [62] and it was found that c-Myc, in line with its possible role as a master regulator of the pluripotent state, acts much earlier than the other three factors during the reprogramming process. Furthermore, although c-Myc was found bound together with the other three factors at many promoters in both iPSCs and ESCs, it would seem that promoter occupancy of the other three factors is dependent on an earlier remodelling of the chromatin and occupancy by c-Myc. Cell cycle alterations towards a more PSC-like cell cycle structure also take place during the first steps of reprogramming, and it is tempting to speculate that c-Myc co-ordinates a general transition of both the somatic transcription network and the somatic cell cycle to a PSC-like state. However, in addition to maintenance of the pluripotent state, it has also been reported that *c-Myc* is required for the differentiation of haemopoietic stem cells [82] and human epidermal stem cells [83,84]. Such reports have led to the growing view that the activity of *Myc* is context-dependent and it has been suggested that, rather than controlling a number of genes involved in cell cycle progression and pluripotency, *Myc* instead is a regulator of an epigenetic state which favours pluripotency but responds to the environment of the cell [84], perhaps poising certain genes for activation with bivalent chromatin modifications [85]. This idea is supported further by data demonstrating that Myc assembles into regulatory complexes with both activating and repressive activities [81]. *Myc* regulates a vast number of genes [62,80,85–90], including miRNAs, and in this context it is interesting that some functions of *Myc* can be substituted for by the expression of ESC-specific miRNAs, which may themselves be regulated by *Myc* [91].

The data described above strongly suggest that c-Myc should be essential for the induction of reprogramming. However, this is not the case [92–95]. Reprogramming to full pluripotency has been achieved using only three factors [94,97]. The fact that other genes can substitute for Myc in enhancing the efficiency of reprogramming [70] also suggest that Myc itself is not essential for the process of efficient reprogramming. Although previous reports also claimed reprogramming to pluripotency using only Sox2 [62], a recent study demonstrates that Sox2 overexpression in fibroblasts is sufficient for conversion into a neural stem cell state [98] and so the role of this factor in reprogramming is unclear. However, in the absence of c-Myc, the efficiency of reprogramming is significantly reduced. This is not surprising, as the involvement of c-Myc in down-regulating the somatic cell transcriptional programme places it within the most efficient step of the reprogramming timetable [62]; it appears that the establishment of the pluripotency network is the least efficient step in the process. In summary, it is clear that Myc acts to co-ordinate both the PSC-like cell cycle and the pluripotency transcription network in PSCs, and may have further roles in allowing the transition from a pluripotent state to a differentiated state. Such co-ordination suggests that both processes are necessary for the phenotype of PSCs, but does not preclude direct interaction between the two.

## CONCLUSIONS

**Figure 3:**
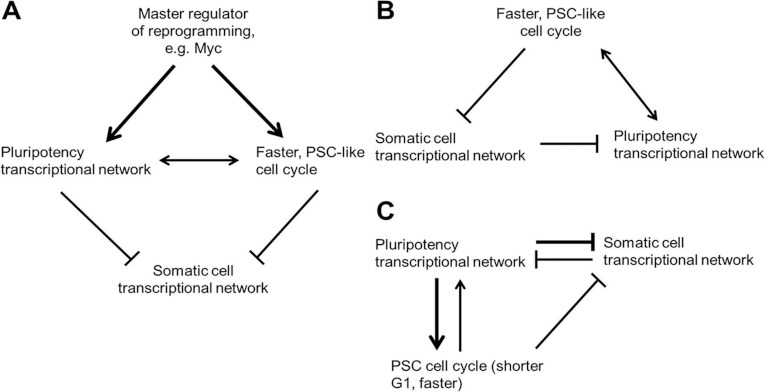
Potential interactions between the cell cycle and the pluripotency transcription network in pluripotent cells and during reprogramming (**A**) A master regulator, such as Myc, regulates both the pluripotency network and the cell cycle, which co-operate to down-regulate the somatic transcription network. (**B**) The pluripotent cell cycle structure stabilizes the pluripotent transcription network and destabilizes the somatic cell transcription network directly. (**C**) The pluripotency transcription network and the cell cycle act in a positive-feedback loop that represses the somatic cell transcription network.

It is clear that the cell cycle and the pluripotent state are intimately connected in PSCs, a fact that has already been exploited to enhance reprogramming efficiency by isolating cells displaying a PSC-like cell cycle early during the reprogramming process [99]. The unusual cell cycle structure and activity of CDKs present in PSCs is remodelled to a more general somatic cell cycle during, or soon after, the loss of pluripotency when PSCs differentiate. The question implicit is one of cause and effect: does the loss of pluripotency cause restructuring of the cell cycle or does restructuring of the cell cycle cause restricted cell fate? From the data available so far it appears that each may be involved in the regulation of each other. Pluripotency factors such as *Nanog* and *Myc* regulate the expression of cell cycle components but, as highlighted in the present review, the general cell cycle structure can also regulate pluripotency. However, whether specific cyclin–CDK complexes have direct roles in the regulation of pluripotency is still unclear, as is whether pluripotency and cell cycle may both be controlled by a master regulator (summarized in Figure 3). It seems likely that a greater understanding of mutual regulation of the cell cycle and the pluripotent state could be exploited in regenerative medicine, utilizing manipulation of the cell cycle to enhance the robustness of somatic cell reprogramming to generate iPSCs. So far, very few studies have sought to apply such techniques or to address such questions. The answers promise to not only drive the clinical application of such technology, but also provide insights into the basic biology of PSCs.

## References

[1] Murray A. W., Hunt T. (1993). The Cell Cycle: an Introduction.

[2] Sherr C. J., Roberts J. M. (1999). CDK inhibitors: positive and negative regulators of G1-phase
progression. Genes Dev..

[3] Stead E., White J., Faast R., Conn S., Goldstone S., Rathjen J., Dhingra U., Rathjen P., Walker D., Dalton S. (2002). Pluripotent cell division cycles are driven by ectopic Cdk2, cyclin A/E and E2F
activities. Oncogene.

[4] Lawson K. A., Meneses J. J., Pedersen R. A. (1991). Clonal analysis of epiblast fate during germ layer formation in the mouse
embryo. Development.

[5] Singh A. M., Dalton S. (2009). The cell cycle and Myc intersect with mechanisms that regulate pluripotency and
reprogramming. Cell Stem Cell.

[6] Ballabeni A., Park I. H., Zhao R., Wang W., Lerou P. H., Daley G. Q., Kirschner M. W. (2011). Cell cycle adaptations of embryonic stem cells. Proc. Natl. Acad. Sci. U.S.A..

[7] White J., Stead E., Faast R., Conn S., Cartwright P., Dalton S. (2005). Developmental activation of the Rb-E2F pathway and establishment of cell
cycle-regulated cyclin-dependent kinase activity during embryonic stem cell
differentiation. Mol. Biol. Cell.

[8] Filipczyk A. A., Laslett A. L., Mummery C., Pera M. F. (2007). Differentiation is coupled to changes in the cell cycle regulatory apparatus of human
embryonic stem cells. Stem Cell Res..

[9] Dalton S. (2009). Exposing hidden dimensions of embryonic stem cell cycle control. Cell Stem Cell.

[10] Ohtsuka S., Dalton S. (2008). Molecular and biological properties of pluripotent embryonic stem
cells. Gene Ther..

[11] Newport J., Kirschner M. (1982). A major developmental transition in early *Xenopus* embryos: I.
characterization and timing of cellular changes at the midblastula stage. Cell.

[12] Saka Y., Smith J. C. (2001). Spatial and temporal patterns of cell division during early *Xenopus*
embryogenesis. Dev. Biol..

[13] Vernon A. E., Philpott A. (2003). A single cdk inhibitor, p27Xic1, functions beyond cell cycle regulation to promote
muscle differentiation in *Xenopus*. Development.

[14] Bloom J., Cross F. R. (2007). Multiple levels of cyclin specificity in cell-cycle control. Nat. Rev..

[15] Santamaria D., Barriere C., Cerqueira A., Hunt S., Tardy C., Newton K., Caceres J. F., Dubus P., Malumbres M., Barbacid M. (2007). Cdk1 is sufficient to drive the mammalian cell cycle. Nature.

[16] Sherr C. J., Roberts J. M. (2004). Living with or without cyclins and cyclin-dependent kinases. Genes Dev..

[17] Hartley R. S., Rempel R. E., Maller J. L. (1996). *In vivo* regulation of the early embryonic cell cycle in
*Xenopus*. Dev. Biol..

[18] Buecker C., Geijsen N. (2010). Different flavors of pluripotency, molecular mechanisms, and practical
implications. Cell Stem Cell.

[19] Ben-David U., Kopper O., Benvenisty N. (2012). Expanding the boundaries of embryonic stem cells. Cell Stem Cell.

[20] Nichols J., Smith A. (2009). Naive and primed pluripotent states. Cell Stem Cell.

[21] Macfarlan T. S., Gifford W. D., Driscoll S., Lettieri K., Rowe H. M., Bonanomi D., Firth A., Singer O., Trono D., Pfaff S. L. (2012). Embryonic stem cell potency fluctuates with endogenous retrovirus
activity. Nature.

[22] Van Hoof D., Munoz J., Braam S. R., Pinkse M. W., Linding R., Heck A. J., Mummery C. L., Krijgsveld J. (2009). Phosphorylation dynamics during early differentiation of human embryonic stem
cells. Cell Stem Cell.

[23] Maimets T., Neganova I., Armstrong L., Lako M. (2008). Activation of p53 by nutlin leads to rapid differentiation of human embryonic stem
cells. Oncogene.

[24] Gross I., Lhermitte B., Domon-Dell C., Duluc I., Martin E., Gaiddon C., Kedinger M., Freund J. N. (2005). Phosphorylation of the homeotic tumor suppressor Cdx2 mediates its
ubiquitin-dependent proteasome degradation. Oncogene.

[25] Neganova I., Zhang X., Atkinson S., Lako M. (2009). Expression and functional analysis of G1 to S regulatory components reveals an
important role for CDK2 in cell cycle regulation in human embryonic stem
cells. Oncogene.

[26] Deshpande A. M., Dai Y. S., Kim Y., Kim J., Kimlin L., Gao K., Wong D. T. (2009). Cdk2ap1 is required for epigenetic silencing of Oct4 during murine embryonic stem
cell differentiation. J. Biol. Chem..

[27] Kim Y., McBride J., Kimlin L., Pae E. K., Deshpande A., Wong D. T. (2009). Targeted inactivation of p12, CDK2 associating protein 1, leads to early embryonic
lethality. PLoS ONE.

[28] Zhang X., Neganova I., Przyborski S., Yang C., Cooke M., Atkinson S. P., Anyfantis G., Fenyk S., Keith W. N., Hoare S. F. (2009). A role for NANOG in G1 to S transition in human embryonic stem cells through direct
binding of CDK6 and CDC25A. J. Cell Biol..

[29] Savatier P., Lapillonne H., Jirmanova L., Vitelli L., Samarut J. (2002). Analysis of the cell cycle in mouse embryonic stem cells. Methods Mol. Biol..

[30] Madine M., Laskey R. (2001). Geminin bans replication licence. Nat. Cell Biol..

[31] McGarry T. J., Kirschner M. W. (1998). Geminin, an inhibitor of DNA replication, is degraded during mitosis. Cell.

[32] Seo S., Kroll K. L. (2006). Geminin's double life: chromatin connections that regulate transcription at the
transition from proliferation to differentiation. Cell Cycle.

[33] Gonzalez M. A., Tachibana K. E., Adams D. J., van der Weyden L., Hemberger M., Coleman N., Bradley A., Laskey R. A. (2006). Geminin is essential to prevent endoreduplication and to form pluripotent cells
during mammalian development. Genes Dev..

[34] Yang V. S., Carter S. A., Hyland S. J., Tachibana-Konwalski K., Laskey R. A., Gonzalez M. A. (2011). Geminin escapes degradation in G1 of mouse pluripotent cells and mediates the
expression of Oct4, Sox2, and Nanog. Curr. Biol..

[35] Ohno Y., Yasunaga S., Ohtsubo M., Mori S., Tsumura M., Okada S., Ohta T., Ohtani K., Kobayashi M., Takihara Y. (2010). Hoxb4 transduction down-regulates Geminin protein, providing hematopoietic stem and
progenitor cells with proliferation potential. Proc. Natl. Acad. Sci. U.S.A..

[36] Shinnick K. M., Eklund E. A., McGarry T. J. (2010). Geminin deletion from hematopoietic cells causes anemia and thrombocytosis in
mice. J. Clin. Invest..

[37] Gaspar-Maia A., Alajem A., Meshorer E., Ramalho-Santos M. (2011). Open chromatin in pluripotency and reprogramming. Nat. Rev. Mol. Cell Biol..

[38] Meissner A., Mikkelsen T. S., Gu H., Wernig M., Hanna J., Sivachenko A., Zhang X., Bernstein B. E., Nusbaum C., Jaffe D. B. (2008). Genome-scale DNA methylation maps of pluripotent and differentiated
cells. Nature.

[39] Bernstein B. E., Mikkelsen T. S., Xie X., Kamal M., Huebert D. J., Cuff J., Fry B., Meissner A., Wernig M., Plath K. (2006). A bivalent chromatin structure marks key developmental genes in embryonic stem
cells. Cell.

[40] Efroni S., Duttagupta R., Cheng J., Dehghani H., Hoeppner D. J., Dash C., Bazett-Jones D. P., Le Grice S., McKay R. D., Buetow K. H. (2008). Global transcription in pluripotent embryonic stem cells. Cell Stem Cell.

[41] Lavoie G., St-Pierre Y. (2011). Phosphorylation of human DNMT1: implication of cyclin-dependent
kinases. Biochem. Biophys. Res. Commun..

[42] Hale T. K., Contreras A., Morrison A. J., Herrera R. E. (2006). Phosphorylation of the linker histone H1 by CDK regulates its binding to
HP1α. Mol. Cell.

[43] Terme J. M., Sesé B., Millán-Ariño L., Mayor R., Izpisúa Belmonte J. C., Barrero M. J., Jordan A. (2011). Histone H1 variants are differentially expressed and incorporated into chromatin
during differentiation and reprogramming to pluripotency. J. Biol. Chem..

[44] Brown D. T. (2003). Histone H1 and the dynamic regulation of chromatin function. Biochem. Cell Biol..

[45] Zhang Y., Cooke M., Panjwani S., Cao K., Krauth B., Ho P. Y., Medrzycki M., Berhe D. T., Pan C., McDevitt T. C. (2012). Histone H1 depletion impairs embryonic stem cell differentiation. PLoS Genet..

[46] Freedman B. S., Heald R. (2010). Functional comparison of H1 histones in *Xenopus* reveals
isoform-specific regulation by Cdk1 and RanGTP. Curr. Biol..

[47] Osley M. A. (1991). The regulation of histone synthesis in the cell cycle. Annu. Rev. Biochem..

[48] Smith S., Stillman B. (1991). Stepwise assembly of chromatin during DNA replication
*in vitro*. EMBO J..

[49] Philpott A., Krude T., Laskey R. A. (2000). Nuclear chaperones. Semin. Cell Dev. Biol..

[50] Verreault A. (2000). *De novo* nucleosome assembly: new pieces in an old
puzzle. Genes Dev..

[51] Petruk S., Sedkov Y., Johnston D. M., Hodgson J. W., Black K. L., Kovermann S. K., Beck S., Canaani E., Brock H. W., Mazo A. (2012). TrxG and PcG proteins but not methylated histones remain associated with DNA through
replication. Cell.

[52] Aoto T., Saitoh N., Sakamoto Y., Watanabe S., Nakao M. (2008). Polycomb group protein-associated chromatin is reproduced in post-mitotic G1 phase
and is required for S phase progression. J. Biol. Chem..

[53] Laskey R. A., Gurdon J. B. (1970). Genetic content of adult somatic cells tested by nuclear transplantation from
cultured cells. Nature.

[54] Briggs R., King T. J. (1952). Transplantation of living nuclei from blastula cells into enucleated frogs’
eggs. Proc. Natl. Acad. Sci. U.S.A..

[55] Gurdon J. B., Elsdale T. R., Fischberg M. (1958). Sexually mature individuals of *Xenopus laevis* from the
transplantation of single somatic nuclei. Nature.

[56] Takahashi K., Yamanaka S. (2006). Induction of pluripotent stem cells from mouse embryonic and adult fibroblast
cultures by defined factors. Cell.

[57] Buganim Y., Faddah D. A., Cheng A. W., Itskovich E., Markoulaki S., Ganz K., Klemm S. L., van Oudenaarden A., Jaenisch R. (2012). Single-cell expression analyses during cellular reprogramming reveal an early
stochastic and a late hierarchic phase. Cell.

[58] Hanna J., Saha K., Pando B., van Zon J., Lengner C. J., Creyghton M. P., van Oudenaarden A., Jaenisch R. (2009). Direct cell reprogramming is a stochastic process amenable to
acceleration. Nature.

[59] Stadtfeld M., Maherali N., Breault D. T., Hochedlinger K. (2008). Defining molecular cornerstones during fibroblast to iPS cell reprogramming in
mouse. Cell Stem Cell.

[60] Huangfu D., Maehr R., Guo W., Eijkelenboom A., Snitow M., Chen A. E., Melton D. A. (2008). Induction of pluripotent stem cells by defined factors is greatly improved by
small-molecule compounds. Nat. Biotechnol..

[61] Maherali N., Sridharan R., Xie W., Utikal J., Eminli S., Arnold K., Stadtfeld M., Yachechko R., Tchieu J., Jaenisch R. (2007). Directly reprogrammed fibroblasts show global epigenetic remodeling and widespread
tissue contribution. Cell Stem Cell.

[62] Sridharan R., Tchieu J., Mason M. J., Yachechko R., Kuoy E., Horvath S., Zhou Q., Plath K. (2009). Role of the murine reprogramming factors in the induction of
pluripotency. Cell.

[63] Hanna J., Markoulaki S., Schorderet P., Carey B. W., Beard C., Wernig M., Creyghton M. P., Steine E. J., Cassady J. P., Foreman R. (2008). Direct reprogramming of terminally differentiated mature B lymphocytes to
pluripotency. Cell.

[64] Cobaleda C., Jochum W., Busslinger M. (2007). Conversion of mature B cells into T cells by dedifferentiation to uncommitted
progenitors. Nature.

[65] Weintraub H., Tapscott S. J., Davis R. L., Thayer M. J., Adam M. A., Lassar A. B., Miller A. D. (1989). Activation of muscle-specific genes in pigment, nerve, fat, liver, and fibroblast
cell lines by forced expression of MyoD. Proc. Natl. Acad. Sci. U.S.A..

[66] Warren L., Manos P. D., Ahfeldt T., Loh Y. H., Li H., Lau F., Ebina W., Mandal P. K., Smith Z. D., Meissner A. (2010). Highly efficient reprogramming to pluripotency and directed differentiation of human
cells with synthetic modified mRNA. Cell Stem Cell.

[67] Ganier O., Bocquet S., Peiffer I., Brochard V., Arnaud P., Puy A., Jouneau A., Feil R., Renard J. P., Méchali M. (2011). Synergic reprogramming of mammalian cells by combined exposure to mitotic
*Xenopus* egg extracts and transcription factors. Proc. Natl. Acad. Sci. U.S.A..

[68] Egli D., Birkhoff G., Eggan K. (2008). Mediators of reprogramming: transcription factors and transitions through
mitosis. Nat. Rev. Mol. Cell Biol..

[69] Takahashi K., Tanabe K., Ohnuki M., Narita M., Ichisaka T., Tomoda K., Yamanaka S. (2007). Induction of pluripotent stem cells from adult human fibroblasts by defined
factors. Cell.

[70] Edel M. J., Menchon C., Menendez S., Consiglio A., Raya A., Izpisua Belmonte J. C. (2010). Rem2 GTPase maintains survival of human embryonic stem cells as well as enhancing
reprogramming by regulating p53 and cyclin D1. Genes Dev..

[71] Medina R., Ghule P. N., Cruzat F., Barutcu A. R., Montecino M., Stein J. L., van Wijnen A. J., Stein G. S. (2012). Epigenetic control of cell cycle-dependent histone gene expression is a principal
component of the abbreviated pluripotent cell cycle. Mol. Cell. Biol..

[72] Li H., Collado M., Villasante A., Strati K., Ortega S., Cañamero M., Blasco M. A., Serrano M. (2009). The Ink4/Arf locus is a barrier for iPS cell reprogramming. Nature.

[73] Kawamura T., Suzuki J., Wang Y. V., Menendez S., Morera L. B., Raya A., Wahl G. M., Izpisúa Belmonte J. C. (2009). Linking the p53 tumour suppressor pathway to somatic cell
reprogramming. Nature.

[74] Ruiz S., Panopoulos A. D., Herrerías A., Bissig K. D., Lutz M., Berggren W. T., Verma I. M., Izpisua Belmonte J. C. (2011). A high proliferation rate is required for cell reprogramming and maintenance of human
embryonic stem cell identity. Curr. Biol..

[75] Agami R., Bernards R. (2000). Distinct initiation and maintenance mechanisms cooperate to induce G1 cell cycle
arrest in response to DNA damage. Cell.

[76] Meyer N., Penn L. Z. (2008). Reflecting on 25 years with MYC. Nat. Rev. Cancer.

[78] Hanna J., Markoulaki S., Mitalipova M., Cheng A. W., Cassady J. P., Staerk J., Carey B. W., Lengner C. J., Foreman R., Love J. (2009). Metastable pluripotent states in NOD-mouse-derived ESCs. Cell Stem Cell.

[79] Baudino T. A., McKay C., Pendeville-Samain H., Nilsson J. A., Maclean K. H., White E. L., Davis A. C., Ihle J. N., Cleveland J. L. (2002). c-Myc is essential for vasculogenesis and angiogenesis during development and tumor
progression. Genes Dev..

[80] Smith K. N., Singh A. M., Dalton S. (2010). Myc represses primitive endoderm differentiation in pluripotent stem
cells. Cell Stem Cell.

[81] Smith K. N., Lim J. M., Wells L., Dalton S. (2011). Myc orchestrates a regulatory network required for the establishment and maintenance
of pluripotency. Cell Cycle.

[82] Wilson A., Murphy M. J., Oskarsson T., Kaloulis K., Bettess M. D., Oser G. M., Pasche A. C., Knabenhans C., Macdonald H. R., Trumpp A. (2004). c-Myc controls the balance between hematopoietic stem cell self-renewal and
differentiation. Genes Dev..

[83] Lo Celso C., Berta M. A., Braun K. M., Frye M., Lyle S., Zouboulis C. C., Watt F. M. (2008). Characterization of bipotential epidermal progenitors derived from human sebaceous
gland: contrasting roles of c-Myc and β-catenin. Stem Cells.

[84] Watt F. M., Frye M., Benitah S. A. (2008). MYC in mammalian epidermis: how can an oncogene stimulate
differentiation?. Nat. Rev. Cancer.

[85] Lin C. H., Lin C., Tanaka H., Fero M. L., Eisenman R. N. (2009). Gene regulation and epigenetic remodeling in murine embryonic stem cells by
c-Myc. PLoS ONE.

[86] Fernandez P. C., Frank S. R., Wang L., Schroeder M., Liu S., Greene J., Cocito A., Amati B. (2003). Genomic targets of the human c-Myc protein. Genes Dev..

[87] Guccione E., Martinato F., Finocchiaro G., Luzi L., Tizzoni L., Dall’ Olio V., Zardo G., Nervi C., Bernard L., Amati B. (2006). Myc-binding-site recognition in the human genome is determined by chromatin
context. Nat. Cell Biol..

[88] Chen X., Xu H., Yuan P., Fang F., Huss M., Vega V. B., Wong E., Orlov Y. L., Zhang W., Jiang J. (2008). Integration of external signaling pathways with the core transcriptional network in
embryonic stem cells. Cell.

[89] Kim J., Chu J., Shen X., Wang J., Orkin S. H. (2008). An extended transcriptional network for pluripotency of embryonic stem
cells. Cell.

[90] Kidder B. L., Yang J., Palmer S. (2008). Stat3 and c-Myc genome-wide promoter occupancy in embryonic stem
cells. PLoS ONE.

[91] Judson R. L., Babiarz J. E., Venere M., Blelloch R. (2009). Embryonic stem cell-specific microRNAs promote induced pluripotency. Nat. Biotechnol..

[92] Araki R., Hoki Y., Uda M., Nakamura M., Jincho Y., Tamura C., Sunayama M., Ando S., Sugiura M., Yoshida M. A. (2011). Crucial role of c-Myc in the generation of induced pluripotent stem
cells. Stem Cells.

[93] Li H. Y., Chien Y., Chen Y. J., Chen S. F., Chang Y. L., Chiang C. H., Jeng S. Y., Chang C. M., Wang M. L., Chen L. K. (2011). Reprogramming induced pluripotent stem cells in the absence of c-Myc for
differentiation into hepatocyte-like cells. Biomaterials.

[94] Nakagawa M., Koyanagi M., Tanabe K., Takahashi K., Ichisaka T., Aoi T., Okita K., Mochiduki Y., Takizawa N., Yamanaka S. (2008). Generation of induced pluripotent stem cells without Myc from mouse and human
fibroblasts. Nat. Biotechnol..

[95] Nakagawa M., Takizawa N., Narita M., Ichisaka T., Yamanaka S. (2010). Promotion of direct reprogramming by transformation-deficient Myc. Proc. Natl. Acad. Sci. U.S.A..

[97] Wernig M., Meissner A., Cassady J. P., Jaenisch R. (2008). c-Myc is dispensable for direct reprogramming of mouse fibroblasts. Cell Stem Cell.

[98] Ring K. L., Tong L. M., Balestra M. E., Javier R., Andrews-Zwilling Y., Li G., Walker D., Zhang W. R., Kreitzer A. C., Huang Y. (2012). Direct reprogramming of mouse and human fibroblasts into multipotent neural stem
cells with a single factor. Cell Stem Cell.

[99] Roccio M., Schmitter D., Knobloch M., Okawa Y., Sage D., Lutolf M. P. (2013). Predicting stem cell fate changes by differential cell cycle progression
patterns. Development.

